# COVID-19 in Patients Receiving CD20-depleting Immunochemotherapy for B-cell Lymphoma

**DOI:** 10.1097/HS9.0000000000000603

**Published:** 2021-06-28

**Authors:** Erik Gaitzsch, Verena Passerini, Elham Khatamzas, Carolin D. Strobl, Maximilian Muenchhoff, Clemens Scherer, Andreas Osterman, Michael Heide, Anna Reischer, Marion Subklewe, Alexandra Leutbecher, Benjamin Tast, Adrian Ruhle, Tobias Weiglein, Stephanie-Susanne Stecher, Hans J. Stemmler, Martin Dreyling, Philipp Girl, Enrico Georgi, Roman Wölfel, Laura Mateyka, Elvira D’Ippolito, Kilian Schober, Dirk H. Busch, Juliane Kager, Christoph D. Spinner, Matthias Treiber, Sebastian Rasch, Tobias Lahmer, Roman Iakoubov, Jochen Schneider, Ulrike Protzer, Christof Winter, Jürgen Ruland, Michael Quante, Oliver T. Keppler, Michael von Bergwelt-Baildon, Johannes Hellmuth, Oliver Weigert

**Affiliations:** 1 Department of Medicine III, University Hospital, LMU Munich, Germany; 2 Max von Pettenkofer Institute & Gene Center, Virology, National Reference Center for Retroviruses, LMU München, Germany; 3 German Center for Infection Research (DZIF), partner site Munich, Germany; 4 COVID-19 Registry of the LMU Munich (CORKUM), University Hospital, LMU Munich, Germany; 5 Department of Medicine I, University Hospital, LMU Munich, Germany; 6 Gene Center, Laboratory for Translational Cancer Immunology, Ludwig-Maximilians-Universität München, Germany; 7 German Cancer Consortium (DKTK), Munich, Germany; German Cancer Research Center (DKFZ), Heidelberg, Germany; 8 Department of Medicine II, University Hospital, LMU Munich, Germany; 9 Bundeswehr Institute of Microbiology, Munich, Germany; 10 Institute for Medical Microbiology, Immunology and Hygiene, Technische Universität München, Germany; 11 Technical University of Munich, School of Medicine, University Hospital Rechts der Isar, Department of Internal Medicine II, Munich, Germany; 12 Technical University of Munich, School of Medicine, University Hospital Rechts der Isar, Munich, Germany; 13 Institute of Virology, Technical University of Munich/Helmholtz Zentrum München, Germany; 14 Institute of Clinical Chemistry and Pathobiochemistry, School of Medicine, Technical University of Munich, Germany; 15 Department of Medicine II (Gastroenterology, Hepatology, Endocrinology and Infectious Diseases), Freiburg University Medical Center, Faculty of Medicine, University of Freiburg, Germany

## Abstract

The clinical and immunological impact of B-cell depletion in the context of coronavirus disease 2019 (COVID-19) is unclear. We conducted a prospectively planned analysis of COVID-19 in patients who received B-cell depleting anti-CD20 antibodies and chemotherapy for B-cell lymphomas. The control cohort consisted of age- and sex-matched patients without lymphoma who were hospitalized because of COVID-19. We performed detailed clinical analyses, in-depth cellular and molecular immune profiling, and comprehensive virological studies in 12 patients with available biospecimens. B-cell depleted lymphoma patients had more severe and protracted clinical course (median hospitalization 88 versus 17 d). All patients actively receiving immunochemotherapy (n = 5) required ICU support including long-term mechanical ventilation. Neutrophil recovery following granulocyte colony stimulating factor stimulation coincided with hyperinflammation and clinical deterioration in 4 of the 5 patients. Immune cell profiling and gene expression analysis of peripheral blood mononuclear cells revealed early activation of monocytes/macrophages, neutrophils, and the complement system in B-cell depleted lymphoma patients, with subsequent exacerbation of the inflammatory response and dysfunctional interferon signaling at the time of clinical deterioration of COVID-19. Longitudinal immune cell profiling and functional in vitro assays showed SARS-CoV-2-specific CD8^+^ and CD4^+^ T-effector cell responses. Finally, we observed long-term detection of SARS-CoV-2 in respiratory specimens (median 84 versus 12 d) and an inability to mount lasting SARS-CoV-2 antibody responses in B-cell depleted lymphoma patients. In summary, we identified clinically relevant particularities of COVID-19 in lymphoma patients receiving B-cell depleting immunochemotherapies.

## Introduction

The emergence of severe acute respiratory syndrome coronavirus 2 (SARS-CoV-2) causing coronavirus disease 2019 (COVID-19) has led to a rapidly unfolding pandemic with extensive morbidity and mortality.^1–3^ The clinical presentation of COVID-19 is highly variable, ranging from asymptomatic infection to respiratory failure with fatal outcome.^3–5^ Several risk factors for severe clinical course have been described, including malignancies and immunocompromised state due to anticancer therapies.^2,6–9^ Therefore, clinicians now have to balance the risks and benefit of providing state-of-the-art anticancer therapies and the possibility of more severe clinical course of SARS-CoV-2 infections.^10,11^ However, currently available guidelines are primarily based on expert opinion as data on specific malignancies and treatments is still limited.^12,13^

Standard treatment of patients with B-cell non-Hodgkin lymphomas (B-NHL) frequently includes monoclonal anti-CD20 antibodies (eg, rituximab and obinutuzumab), which deplete B-cells for at least 6–9 months.^14,15^ B-cell depletion could compromise adaptive antiviral immune responses, delay viral clearance, and prolong viral shedding^16–18^ and lead to more severe disease course, higher risk of re-infection, and prolonged infectivity.^19–23^

Here, we report a prospectively planned analysis of COVID-19 in patients from two university hospital registries who received B-cell depleting immunochemotherapies for B-NHL during the first wave of the COVID-19 pandemic in Europe. We provide a detailed analysis of the clinical course with a long-term follow-up along with in-depth profiling of the humoral, cellular and molecular immune responses in comparison to an age-and sex-matched cohort of COVID-19 patients without B-NHL.

## Materials and methods

### Patients and data

**Figure 1.**
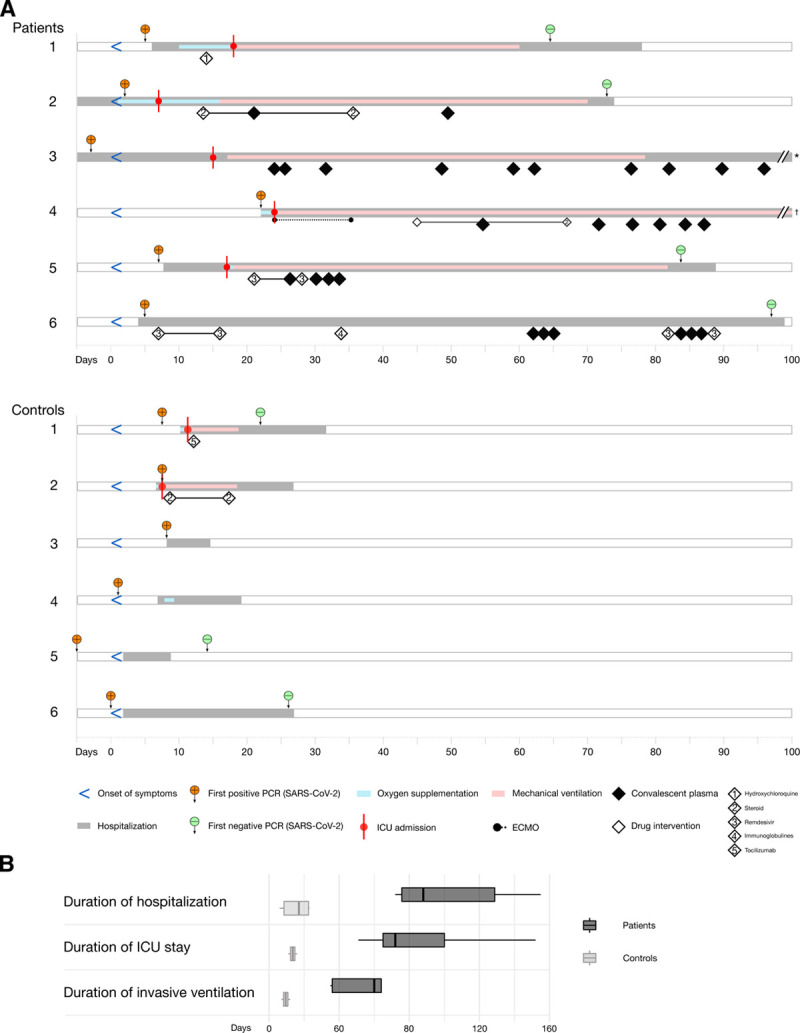
**Severe clinical course of COVID-19 in B-cell depleted lymphoma patients.** (A), Schematic overview of clinical course of COVID-19 in B-cell depleted lymphoma (“patients”, top panel) and COVID-19 patients non-B-cell depleted patients without lymphoma (“controls”, bottom panel). *Discharge from hospital at day 140. **†**Deceased at day 155. (B), Comparison of median durations of hospital stay, ICU stay and mechanical ventilation.

**Figure 2.**
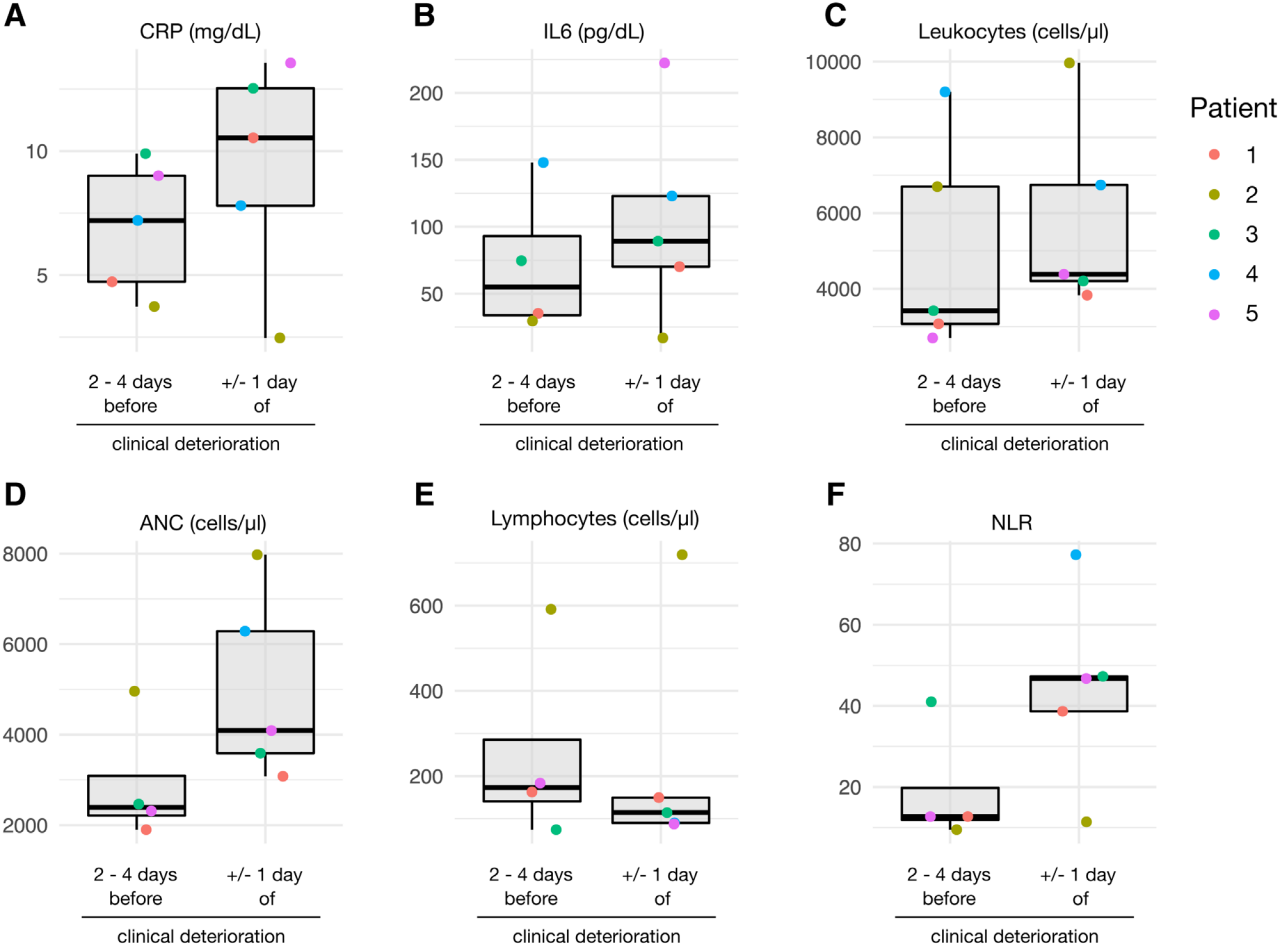
**Hyperinflammation and neutrophil recovery at time of clinical deterioration in B-cell depleted lymphoma patients.** Serum levels of (A) C-reactive protein and (B) interleukin-6 (IL-6), (C) blood leukocytes, (D) absolute neutrophil count (ANC), and (E) lymphocyte counts before and at the time of clinical deterioration. (F), Neutrophil-to-lymphocyte ratio (NLR) before and at the time of clinical deterioration.

Patients are part of the prospective COVID-19 registries of the University Hospital of the Ludwig-Maximilians-University (CORKUM, WHO Trial ID DRKS00021225) or the University Hospital of Technical University of Munich (COMRI). Patients were eligible for this study if they had SARS-CoV-2 infection confirmed by specific real-time polymerase chain reaction (RT-PCR) in a respiratory specimen. The study cohort consisted of patients who received B-cell depleting anti-CD20 antibody-containing immunochemotherapies (ICT) for B-NHL. The control cohort consisted of age- and sex-matched patients without B-NHL who were hospitalized because of symptomatic COVID-19 (See Supplemental Digital Figure 1, http://links.lww.com/HS/A169, CONSORT diagram). We only included patients with available peripheral blood samples (collected centrally up to twice weekly as an optional part of the registries) to determine humoral, cellular and molecular immune profiles (See Supplemental Digital Figure 2, http://links.lww.com/HS/A169). Clinical and routine laboratory data were prospectively collected within the COVID-19 registries and verified and complemented by individual chart review. Clinical deterioration was defined as the need of ICU support. Respiratory deterioration was defined as the need of mechanical ventilation.

Patient data were pseudonymized for analysis, and the study was approved by the local ethics committees (No: #20-245 and #221/20 S). Written informed consent was obtained from each patient or authorized by proxy before any study-related procedure.

### SARS-CoV-2 RT-PCR and serology

RT-PCR from respiratory specimen and blood serum used targets in the N and E genes as previously described.^24^ IgG against SARS-CoV-2 was detected in serum samples using Anti-SARS-CoV-2-ELISA targeting the spike domain S1, according to manufacturers’ protocols (Euroimmune and Roche).^25^ Additional technical details are provided in Supplemental Methods section, http://links.lww.com/HS/A169.

### Virus culture

Culture for SARS-CoV-2 was performed as previously described.^26^ In brief, Vero E6 cells were inoculated with patient respiratory samples (nasopharygeal swap or tracheal aspirate as indicated) and observed daily for a cytopathic effect up to day 7. If negative, an additional passage was performed and observed for another 7 days. On observation of a cytopathogenic effect, the supernatant was harvested and further analyzed for the presence of SARS-CoV-2 RNA by specific real-time PCR detecting the N gene.

### Flow cytometry of peripheral blood mononuclear cells

Immune profiling of peripheral blood mononuclear cells (PBMCs) was done by 32-parameter flow cytometry according to previously published guidelines.^27^ Flow cytometry measurements were performed on a CytoFLEX LX flow cytometer (Beckman Coulter) and data were analyzed using FlowJo, v10 (Tree Star Inc., Ashland, OR, USA). The commercial fluorochrome conjugated antibodies are listed in the Supplemental Methods, http://links.lww.com/HS/A169.

### Ex vivo analysis of SARS-CoV-2-specific T-cell responses

Ex vivo analysis was performed as detailed in the Supplemental Methods section, http://links.lww.com/HS/A169. Briefly, PBMCs where incubated with SARS-CoV-2 peptide pools (Miltenyi Biotec, Bergisch Gladbach, Germany), stained for T-cell surface antigens and intracellular cytokines (including IFNγ) and analyzed by flow cytometry.

### Digital multiplexed gene expression profiling of PBMCs

Digital multiplexed gene expression profiling of PBMCs was performed as previously described.^28,29^ Briefly, RNA from PBMCs was isolated using the Qiagen RNeasy Mini Kit (Qiagen, Hilden, Germany). RNA concentration was measured by NanoDrop 1000 (ThermoFisher, Waltham, MA, USA) and 100 ng were assayed with the PanCancer Human IO 360 Panel according to the manufacturer’s protocol (NanoString, Seattle, WA, USA). Normalization was performed by subtracting the mean + 2 SDs of negative control as a cutoff and adjustment for geometric mean of defined positive controls and defined housekeeping genes. Housekeeping gene normalization threshold was set at 0.1- to 10-fold.

All data and protocols are available to other investigators. For original data, please contact oliver.weigert@med.uni-muenchen.de.

## Results

We enrolled a total of 12 patients who were hospitalized consecutively between March 26 and May 6, 2020 because of symptomatic COVID-19 confirmed by SARS-CoV-2-specific RT-PCR. Table 1 summarizes the patient and disease characteristics. The study cohort consisted of 6 patients (median age 69 yrs, range 43–72) who had received either rituximab- (R, n = 4) or obinutuzumab- (O, n = 2) based immunochemotherapy (ICT) as frontline treatment for follicular lymphoma (n = 3), diffuse large B-cell lymphoma (n = 2), or marginal-zone lymphoma (n = 1). Five patients (no. 1–5) were on active frontline treatment. Patients no. 1–5 had received R/O-CHOP, while patient no. 6 had completed R-Bendamustine ICT 5 months before her SARS-CoV-2 infection. The control cohort consisted of 6 age- and sex-matched COVID-19 patients with no history of B-NHL or B-cell depleting therapies (Table 1).

**Table 1.** Patient and Disease Characteristics, Clinical Course, and Treatment

Patient Characteristics	B-cell Depleted Lymphoma Patients (n = 6)	Controls: non-B-cell Depleted Patients (n = 6)
Age median (yrs) (range)	69 (43–72)	67 (40–75)
Male sex	2	3
B-cell lymphoma	6	0
- Follicular lymphoma	3	n/a
- DLBCL	2	n/a
- Marginal zone lymphoma	1	n/a
B-cell depleting immunochemotherapy	6	0
- Active treatment	5	n/a
- Rituximab-based	4	n/a
• CHOP	3	
• Bendamustine	1	
- Obinutuzumab-based	2	n/a
• CHOP	2	
Risk factor for severe COVID-19		
- Arterial hypertension	2	4
- Diabetes	2	2
- Chronic lung disease	0	1
- Obesity	1	1
ICU admission	5	2
Mechanical ventilation	5	2
Hospital stay median (d) (range)	88 (72–155)	17 (7–23)
ICU stay median (d) (range)	68 (51–155)	14 (11–16)
Mechanical ventilation median (d) (range)	62 (35–152)	10 (7–12)
Death	1	0
COVID-19 directed therapies		
- Remdesivir	2	0
- Convalescent plasma	5	0
- High dose steroids	2	1
- Hydroxychloroquine	1	0
- Tocilizumab	0	1
- Immunoglobulins	1	0
Renal replacement therapy	3	0
Extracorporeal life support	1	0
Prone positioning	2	1
Empiric antibiotic therapy	6	3
Infections	6	3
- Bacterial co-infection	3	0
Central line infection	1	
Urinary tract infection	2	
- Viral co-infection	4	0
HSV stomatitis	2	
HSV pneumonia	1	
CMV reactivation	1	
- Fungal co-infection	2	0
Oral candidiasis	1	
Candida pneumonia	1	

We compared the known clinical risk factors for severe COVID-19 manifestation between B-cell depleted lymphoma patients and controls (Table 1): 1 and 4 patients had arterial hypertension, 2 each had diabetes, 0 and 1 had chronic pulmonary disease, and 1 each was obese, respectively.

### Patients undergoing B-cell depleting ICT had more severe and protracted clinical course

Figure 1A summarizes the clinical course of all patients. The median hospitalization time was 88 days (range 72–155) for B-cell depleted lymphoma patients as compared to 17 days (range 6–23) for controls (Fig. 1B). All lymphoma patients received COVID-19-directed therapies, including remdesivir (n = 2), high-dose steroids (n = 2), hydroxychloroquine (n = 1), immunoglobulins (n = 1), and COVID-19-convalescent plasma (n = 5). In addition, all lymphoma patients received antimicrobial therapies (Table 1). In the control cohort, only 2 patients received COVID-19-directed therapies (high-dose steroids, n = 1, and tocilizumab, n = 1).

All but 1 lymphoma patient (83%) required mechanical ventilation as compared to 2 control patients (33%). Respiratory failure developed in all lymphoma patients on active ICT (no. 1–5) within a median of 14 days (range 11–26 d) after the diagnosis of COVID-19, while patient no. 6 (who had completed ICT before her SARS-CoV-2 infection) had a remarkably mild clinical course. The median time on mechanical ventilation was 62 versus 10 days, the median ICU stay 68 versus 14 days. ICU management of B-cell depleted lymphoma patients was highly complex including extracorporeal membrane oxygenation (n = 1), continuous veno-venous hemodialysis (n = 3), and extracorporeal cytokine adsorption (CytoSorb, n = 1). After a median follow-up of 169 days (range 81–217), 5 patients were alive and discharged from the hospital, while 1 patient (no. 4) died from multiorgan failure on day 155 of her ICU stay after a consensus decision was made to discontinue life-sustaining care.

### Neutrophil recovery frequently coincided with hyperinflammation and clinical deterioration

Overall, lymphoma patients had rising inflammatory markers in routine laboratory testing at the time of clinical deterioration, including C-reactive protein (CRP) and IL-6 serum levels (Fig. 2A, B). Respiratory deterioration coincided with leukocyte and neutrophil recovery (Fig. 2C, D) and a trend toward lower lymphocyte counts (Fig. 2E), resulting in a massively increased neutrophil-lymphocyte-ratio (NLR, Fig. 2F). Of note, 4 of 5 patients on active ICT had received granulocyte colony stimulating factor (G-CSF) injections to accelerate neutrophil recovery, there of 3 within 1 week before ICU admission. G-CSF was given as part of the R-CHOP14 protocol^30^ in 2 patients (no. 2 and 4) or to treat chemotherapy-induced neutropenia in another 2 patients (no. 1 and 3) for 3 and 4 days, respectively (see Supplemental Digital Table 1, http://links.lww.com/HS/A169).

### B-cell depleted patients can mount a SARS-CoV-2-specific T-effector cell response

**Figure 3.**
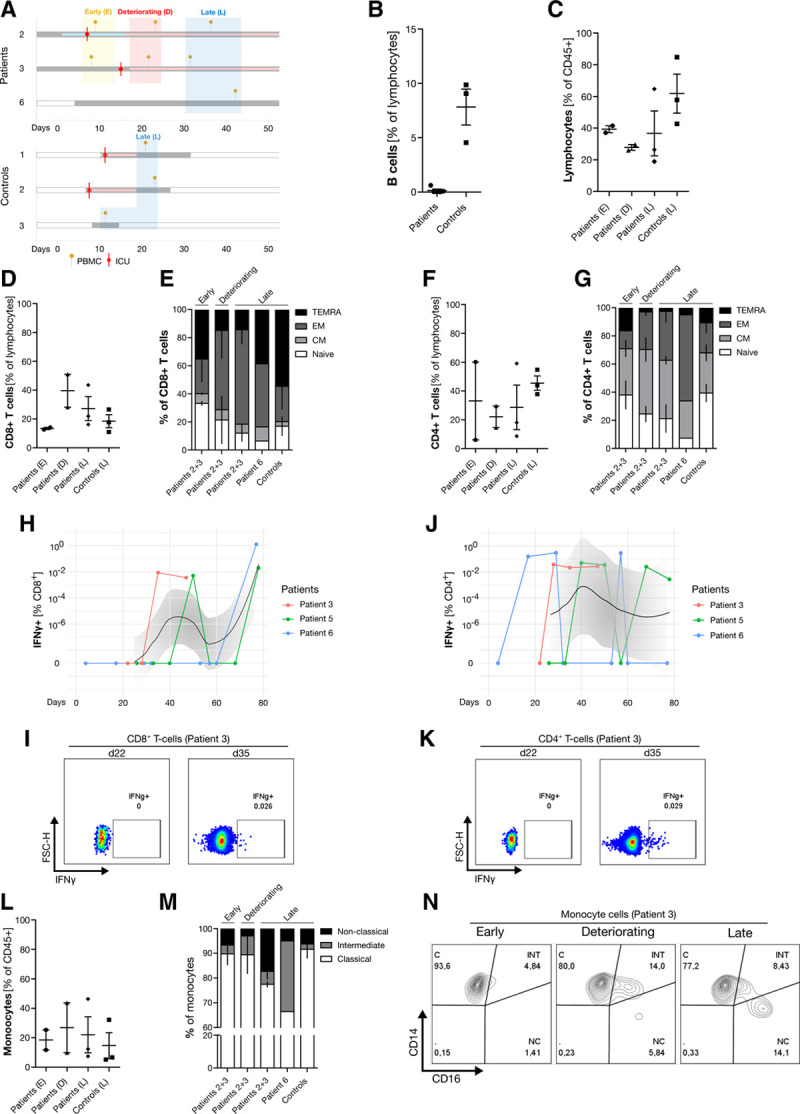
**B-cell depletion, T-cell and monocyte response in B-cell depleted lymphoma patients and controls.** (A), Schematic overview of sampling and timeline. Red mark indicates ICU admission; yellow pins indicate time of PBMC collection. (B), Percentage of B-cells in patients and controls (all time points). (C), Percentage of lymphocytes in PBMCs as indicated in (A). (D), Percentage of lymphocytes and CD8^+^ T-cells in PBMCs as indicated in (A). (E), CD8^+^ T-cell subsets in PBMCs as indicated in (A). (F), Percentage of lymphocytes and CD4^+^ T-cells in PBMCs as indicated in (A). (G), CD4^+^ T-cell subsets as indicated in (A). (H), Percentage of IFNγ-positive CD8^+^ T-cells after ex vivo stimulation with SARS-CoV-2 spike protein. Shown are results of patients with available serial blood samples (n > 3) with interpolation line (black) and 95% confidence interval (gray). (I), Exemplary FACS dot plot for IFNγ-positive CD8^+^ T-cells from patient 3 at indicated time points. (J), Percentage of IFNγ-positive CD4^+^ T-cells after ex vivo stimulation with SARS-CoV-2 spike protein. Shown are results of patients with available serial blood samples (n > 3) with interpolation line (black) and 95% confidence interval (gray). (K), Exemplary FACS dot plot for IFNγ-positive CD4^+^ T-cells from patient 3 at indicated time points. (L), Percentage of monocytes in PBMCs as indicated in (A). (M), Monocyte subsets in PBMCs as indicated in (A). (N), Exemplary FACS contour plot showing monocyte subsets from patient 3 at indicated time points. C = classical; CM = central memory; D = deteriorating; E = early; EM = effector memory; INT = intermediate; L = late; N = naive; NC = non-classical; TEMRA = T-effector memory cells re-expressing CD45RA.

We next investigated the adaptive immune response in B-cell depleted SARS-CoV-2-infected patients (Fig. 3A). Flow cytometry of PBMCs confirmed complete B-cell depletion in all lymphoma patients (Fig. 3B). At the time of clinical deterioration, absolute lymphocyte counts dropped further in these patients (Fig. 3C), but the fraction of CD8^+^ T-cells increased (Fig. 3D). Furthermore, we noticed a shift from naïve phenotype to an effector memory (EM) subtype (Fig. 3E). A similar predominance of EM as well as T-effector memory cells re-expressing CD45RA (TEMRA) was also seen in CD8^+^ T-cells from the B-cell depleted lymphoma patient with uncomplicated COVID-19 (Patient no. 6) and in CD8^+^ T-cells from control patients, all collected at later time points of the disease (Fig. 3E). In CD4^+^ T-cells, the central memory (CM) phenotype fraction was generally larger compared to CD8^+^ T-cells, but we still observed a similar shift from naive T-cells to EM phenotype in B-cell depleted lymphoma patients (Fig. 3F, G).

To investigate whether the expanding EM populations comprise bona fide virus-specific T-cells, we probed CD8^+^ and CD4^+^ T-cells from serially collected peripheral blood samples of B-cell depleted patients for their specificity towards SARS-CoV-2 spike protein. Virus-specific T-cells were not detectable at early time points but became detectable during the course of the disease in all 3 evaluable cases. Time to first measurable SARS-CoV-2 reactive T-cells was highly variable, ranging from 35 to 77 days for CD8^+^ T-cells (Fig. 3H, I) and from 17 to 40 days for CD4^+^ T-cells (Fig. 3J, K), respectively.

### Monocyte hyperactivation in B-cell depleted patients

As activation of the monocyte-macrophage axis has been described before in severe COVID-19,^31^ we determined the shift of monocyte phenotypes during the course of the disease (Fig. 3L, M), distinguishing classical (CD14^+^CD16^−^), intermediate (CD14^+^CD16^+^), and nonclassical (CD14^dim^CD16^+^) monocytes (Fig. 3N).^32^ While classical monocytes are critical for the initial inflammatory response, intermediate monocytes express higher levels of proinflammatory cytokines (including IL-1, IL-6, and IL-10),^33^ and nonclassical monocytes exhibit distinct functional properties, including promotion of neutrophil adhesion to inflamed endothelium.^34^ Interestingly, we observed a shift toward intermediate monocytes at the time of clinical deterioration and subsequently toward nonclassical monocytes at later stages of COVID-19 (Fig. 3M, N), which was more pronounced compared to controls.

### Dysregulated molecular immune responses in B-cell depleted lymphoma patients with COVID-19

**Figure 4.**
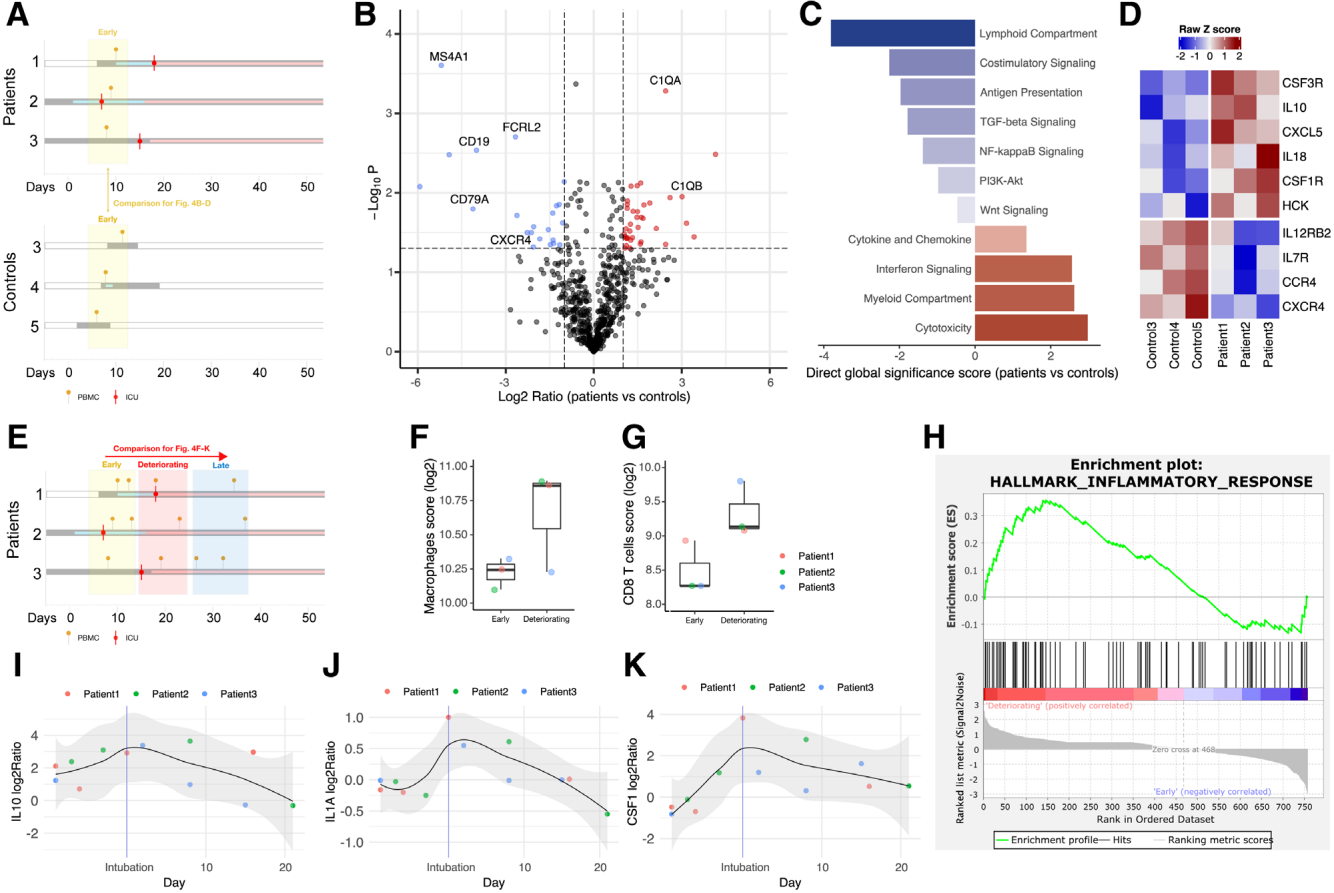
**Dysregulated immune response in B-cell depleted lymphoma patients.** (A), Schematic overview of sampling and timeline for analyses shown in (B) thorough (D). Red mark indicates ICU admission; yellow pin indicates time of PBMC collection. (B), Volcano plot for differential gene expression in patients vs controls (early time points). Genes depicted in red have a *P* value ≤ 0.05 and a log2 ratio higher than 1; genes depicted in blue have a *P* value ≤ 0.05 and a log2 ratio lower than −1. (C), Direct global significant score analysis for each annotated gene set in patients vs controls (at early time points). (D), Heatmap of differentially expressed cytokines and chemokines in patients and controls (at early time points). (E), Schematic overview of longitudinal sampling in patients 1, 2, and 3 for analyses shown in subfigures (F) though (K). Inferred abundance of (F) macrophages and (G) CD8^+^ T-cells (early versus time of clinical deterioration). Changes in the gene expression (log2 ratio) of (I) *IL10*, (J) *IL1A*, and (K) *CSF1* over time. (H), GSEA for the hallmark signature “inflammatory response.”

To comprehensively characterize immune responses in B-cell depleted lymphoma patients versus controls on a transcriptional level, we performed immune gene expression profiling from PBMCs collected at early time points of COVID-19 (ie, before clinical deterioration) (Fig. 4A). Unsupervised clustering clearly separated lymphoma patients from controls (see Supplemental Figure 3A, http://links.lww.com/HS/A169). We inferred abundances of individual PBMC populations by marker gene expression. As expected, B-cell depleted lymphoma patients had lower B-cell scores as compared to controls (see Supplemental Figure 4B, http://links.lww.com/HS/A169), showing significant and large fold lower expression of B-cell genes, including *MS4A1* (*CD20*), *CD19*, *CD79A,* and *FCRL2* (Fig. 4B). Gene set analysis showed downregulation of the “lymphoid compartment” (Fig. 4C), including lower expression of *IL7R*, *CCR4*, and *CXCR4* (Fig. 4D).

In contrast, macrophage and neutrophil scores were higher in B-cell depleted patients as compared to controls (see Supplemental Figure 4C, D, http://links.lww.com/HS/A169). Gene set analysis revealed that top differentially expressed genes were enriched within the biological pathways “cytokine and chemokine signaling,” “interferon signaling,” “myeloid compartment,” and “cytotoxicity,” including upregulation of *IL10, IL18*, *CSF1R,* and *CSF3R* (Fig. 4C, D). Furthermore, differential gene expression showed large fold higher expression of *C1QA* and *C1QB* (Fig. 4B), the first components of the classical serum complement cascade.

**Figure 5.**
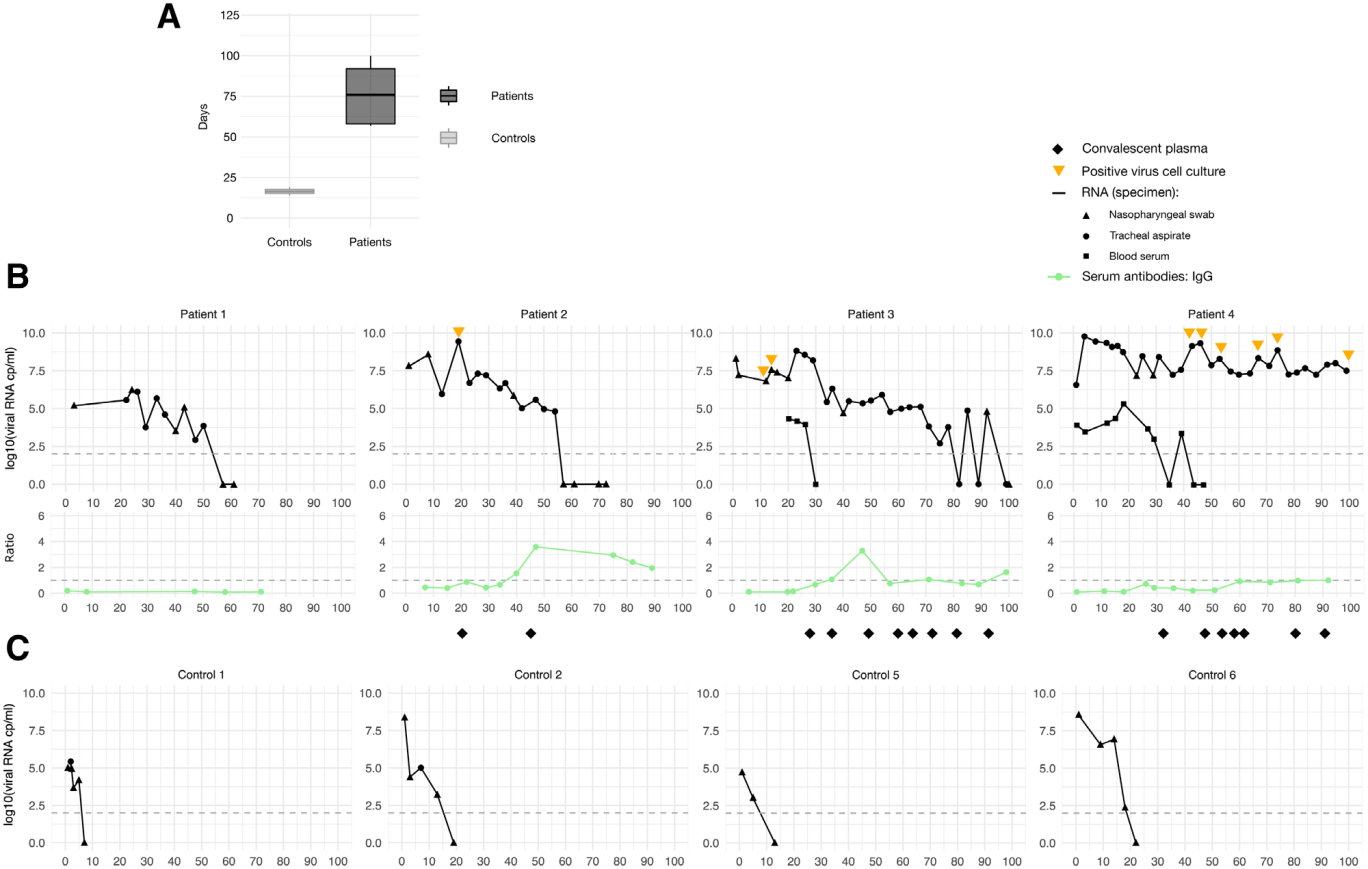
**Delayed viral clearance and impaired SARS-CoV-2 antibody response in B-cell depleted lymphoma patients.** (A), Comparison of median duration of detectable SARS-CoV-2 virus in B-cell depleted lymphoma patients and non-B-cell depleted patients. SARS-CoV-2 viral load in respiratory specimens from nasopharyngeal swabs and tracheal aspiration (black triangle, dots, and lines, respectively), as well as detection of virus in blood serum (black squares) and SARS-CoV-2-specific IgG levels in blood serum in (B) B-cell depleted lymphoma patients, and (C) non-B-cell depleted patients without lymphoma. Viral load is indicated as log10 values of viral RNA copies per milliliter over time per days. Dashed lines indicate the limit of detection for viral load (upper panel) and positivity threshold for IgG levels (lower panel), respectively.

Longitudinal analysis of immune gene expression in PBMCs from B-cell depleted patients (Fig. 4E) showed a further increase in macrophage scores (Fig. 4F) as well as CD8^+^ T-cell scores (Fig. 4G) at the time of clinical deterioration. Gene set enrichment analysis (GSEA) over time showed significant enrichment of the GSEA MSigDB hallmark signature “inflammatory response,” with further upregulation of *IL10*, *IL1A*, and *CSF1* (Fig. 4H-K). Dynamic gene expression analysis revealed patterns with peaks (see Supplemental Digital Figure 5, http://links.lww.com/HS/A169) and troughs (see Supplemnetal Digital Figure 6, http://links.lww.com/HS/A169) at the time of clinical deterioration, including dysregulated interferon-alpha signaling (see Supplemental Digital Figure 4D, http://links.lww.com/HS/A169).

In summary, our immune gene expression indicates early hyperactivation of neutrophils and monocytes/macrophages, as well as the complement system in B-cell depleted patients with COVID-19, with subsequent exacerbation of the inflammatory response and dysfunctional interferon signaling at the time of clinical deterioration.

### Long-term detection of replication-competent SARS-CoV-2 in B-cell depleted lymphoma patients

Finally, we compared SARS-CoV-2 clearance kinetics in patients with and without B-cell depletion. B-cell depleted lymphoma patients had a markedly longer detectability of SARS-CoV-2 RNA in respiratory specimens compared to controls as assessed by RT-PCR (median 84 versus 12 d) (Fig. 5A–C). To determine whether patients shed replication-competent virus, we tested respiratory specimens from 3 lymphoma patients for the presence of virus in cell culture. Remarkably, SARS-CoV-2 could be cultured from specimen of all tested patients, one patient (no. 4) harboring infectious virus for >20 weeks (Fig. 5B). Two patients showed recurring viremia, 1 patient over a time period of >4 weeks. SARS-CoV-2-specific IgG antibodies (Fig. 5B) could only be detected transiently at low levels in 3 B-cell depleted patients shortly after the administration of convalescent plasma. Similar results were obtained with a different antibody assay (see Supplemental Digital Figure 9, http://links.lww.com/HS/A169).

## Discussion

Through detailed analysis of the clinical course and comprehensive laboratory, immunological and virological studies, we identify several clinically relevant particularities of COVID-19 in patients with lymphoma who have received B-cell depleting immunochemotherapies.

First, patients on active immunochemotherapy have long and severe clinical courses. This is in line with earlier reports of severe COVID-19 in B-cell depleted patients.^35,36^ In contrast to the previously reported excess mortality,^37^ only 1 patient in our series died from COVID-19. Of note, this patient presented late during the course of her disease to another hospital before being transferred to our ICU with rapid clinical deterioration, immediately requiring mechanical ventilation and extracorporeal membrane oxygenation support. This suggests that B-cell depleted patients with COVID-19 can benefit from close clinical observation to implement early and rigorous ICU support, including long-term mechanical ventilation.

Second, we observe a coincidence of rapid clinical deterioration and hematological recovery after immunochemotherapy, suggesting that particular close clinical monitoring is mandatory during this critical time period. Neutrophil recovery, further drop of lymphocyte counts, and especially an increasing neutrophil-to-lymphocyte ratio (NLR)^38,39^ may be useful clinical indicators of impending respiratory decompensation.

In fact, the rapid rise of neutrophils and monocytes during hematological recovery could directly contribute to respiratory decompensation by facilitating myeloid cell recruitment to the lungs.^40^ Of note, these cells are highly prevalent in inflammatory lung infiltrates in severe COVID-19.^41^ This may also explain the remarkably indolent clinical course of COVID-19 in our B-cell depleted patient (no. 6) who was not on active lymphoma treatment, and hence, did not undergo this phase of hematologic recovery during her infection. On the other hand, G-CSF is frequently administered to accelerate neutrophil recovery after immunochemotherapy for lymphoma,^30,42,43^ as was the case with 4 of our 5 patients on active treatment. Therefore, our study should at least raise concerns over the routine use of G-CSF in these patients, and justifies further investigations.^44^

Third, our study provides novel insights into the particular immune reactions in response to COVID-19 in B-cell depleted patients. We notice an early activation of the myeloid compartment, with subsequent hyperactivation of monocytes and pathological inflammation with high levels of *IL-6*, *IL-10*, *IL-18*^45^ and others (“cytokine storm”)^46,47^ at the time of hematological recovery and clinical deterioration. However, due to limited size of our study, we realize that this data is primarily hypothesis-generating at this point and requires validation in larger cohorts with longer follow-up.

Finally, our study shows that B-cell depleted patients are incapable of developing lasting SARS-CoV-2 antibody responses, with long-term detectable viral RNA in upper respiratory tract specimens despite receiving convalescent plasma. In fact, we documented shedding of replication-competent virus over months, providing evidence that these patients are potentially capable of transmitting SARS-CoV-2 beyond commonly used quarantine periods. Furthermore, these patients remain at increased risk of SARS-CoV-2 reinfections, as has recently been documented in a case report.^19^ Interestingly, all but 1 B-cell depleted patient ultimately eliminated SARS-CoV-2, yet it took them >6 times longer compared to controls. This strongly suggests that rapid virus elimination critically depends on virus-specific antibody responses.^48–50^ In our series, administration of convalescence plasma resulted only in transient and low antibody levels in some patients and are therefore unlikely to have substantially contributed to virus elimination. Instead, we notice SARS-CoV-2-specific T-effector cell responses over time that are comparable to controls in the resolution phase of COVID-19 (data not shown). It remains to be studied, to what extent these T-cell responses actually contribute to virus elimination and whether they could potentially provide protective immunity in these patients.^51^

We realize that the small cohort size is a major limitation of our study. Also, we only studied patients infected with the SARS-CoV-2 lineages B, B.1, and B.1.1 (data not shown) who were hospitalized during the first wave of the COVID-19 pandemic in Europe. As such, it remains unclear if these results are representative of the clinical course with other SARS-CoV-2 lineages or subsequent pandemic waves in other areas of the world. Importantly, however, our data with long-term follow-up complements previous retrospective studies^9,10,12^ by an in-depth analysis of a homogenous cohort of patients with B-NHL who received B-cell depleting frontline immunochemotherapy. Our major findings are highly consistent and clinically relevant. Based on this study, we now take particular precautions at our institutions in managing COVID-19 in patients who have received B-cell depleting immunochemotherapies:

1. We admit all of these patients to the hospital irrespective of the severity of COVID-19 at the time of presentation.
2. We provide particular close monitoring at the time of hematological recovery and avoid the routine use of G-CSF.
3. We recommend early transfer to an ICU with experience in treating patients with COVID-19, prepared to provide long-term life-sustaining treatment.
4. We monitor these patients for long-term virus excretion and inform patients and their contacts (including health care providers) about the possibility of long-term infectivity and the risk of reinfections.

Ultimately, whenever clinically feasible,^13^ we aim to vaccinate these patients prior to initiating B-cell depleting immunochemotherapies.

## Acknowledgments

We would like to thank all CORKUM/CoMRI investigators and staff. The authors thank the patients and their families for their participation in the CORKUM/CoMRI registry. We acknowledge the iFlow Core Facility of the university hospital Munich (INST 409/225-1 FUGG) for assistance with the generation of flow cytometry data.

## Disclosures

SR received travel grants from Gilead, TL received travel grants from Gilead, Pfizer and MSD. MD received research support from Abbvie, Bayer, Celgene, Janssen, Roche and speakers’ honoraria from Bayer, Celgene, Gilead, Janssen, Roche. MD is part of the scientific advisory board of Astra Zeneca, Bayer, Beigene, Celgene, Gilead, Janssen, Novartis, Roche. All the other authors have no conflicts of interest to disclose.

## Sources of funding

OW was supported by the Deutsche Forschungsgemeinschaft (DFG, German Research Foundation)—Project no. 360372040—SFB 1335. MM and OTK are supported by the BMBF initiative “NaFoUniMedCovid19” (01KX2021), subproject B-FAST and COVIM, and the Bavaria-Saxony research alliance “FOR-COVID.” CORKUM was supported by start-up funds of the LMU.

## Supplementary Material


