# Supplementary material for: COVID-19 in Patients Receiving CD20-depleting Immunochemotherapy for B-cell Lymphoma
Source: Hemasphere. 2021 Jun 28;5(7):e603. doi: 10.1097/HS9.0000000000000603 (PMC8240782; doi:10.1097/HS9.0000000000000603)
Supplement: Supplementary file 1 [file hs9-5-e603-s001.docx]

Supplemental Digital Content (SDC)

Table of content

[1. SDC Methods 2](#_Toc67929820)

[2. SDC Tables 4](#_Toc67929821)

[3. SDC Figures 5](#_Toc67929822)

[4. SDC Reference 14](#_Toc67929823)

# **1. SDC Methods**

*SARS-CoV-2 RT-PCR*

Nucleic acids were extracted from respiratory specimens either using the QIAsymphony DSP Virus/Pathogen Kit or the EZ1 Virus Mini Kit v2.0 (Qiagen, Hilden, Germany). The following PCR assays were used: The nucleocapsid (N1) reaction of the CDC protocol, the envelope amplification of the Charité protocol, the nucleocapsid amplification of the Seegene Allplex 2019-nCoV Assay, the Roche Cobas SARS-CoV-2 nucleocapsid reaction or the Cepheid Xpert Xpress SARS-CoV-2 xx reaction. Viral loads are expressed as SARS-CoV-2-RNA copy numbers per ml sputum, ETA or transport medium of the swab sample (eSwab™, COPAN Diagnostics). Copy numbers are estimated using standard curves that were generated using either a plasmid containing the nucleocapsid gene (2019-nCoV-N-PositiveControl, IDT) or a clinical sample with copy numbers based on digital droplet PCR results as described previously. ^1^ These calculations do not take into account variability between separate PCR runs, different PCR chemicals or different nucleic acid extraction methods.

*Flow cytometry measurement*

The following fluorochrome conjugated monoclonal antibodies CD45 (2D1), CD3 (UCHT-1), CD19 (SJ25C1), CD20 (L27), CD4 (SK3), CD8 (SK1), CD197 (150503), CD45RO (UCHL-1), CD14 (MfP9), CD16 (B73-1) (all from BD Bioscience, Heidelberg, Germany) and CD45 (HI30, Biolegend, San Diego, California) were used. Staining was performed according to manufacturer’s instructions.

*Ex vivo analysis of SARS-CoV-2 specific T-cell responses*

Patient PBMCs were thawed on day -1, washed with warm complete T cell medium (RPMI + 10% FCS + 5% SC+) and rested overnight at ~ 1 million cells/ml (37 °C, 5 % C02). On day 0, 1x10^5^ – 1x10^6^ PBMCs were incubated for 4 h at 37 °C with PepTivator SARS-CoV-2 Protein S (Miltenyi, Cat.Nr.: 130-126-700) (PepTivator Sfinal: 20 µl/ml). DMSO served as negative control and PMA/Ionomycin as positive control. After incubation, cells were stained with Ethidium monoazide (EMA) for live/dead discrimination and subsequently with surface antibodies: CD19-ECD (1:100, Beckman Coulter, Cat. Nr. A07770, clone J3-119), CD8-PE (1:200, Invitrogen, Cat. Nr. MHCD0804, clone 3B5), CD3-BV421 (1:100, BD Bioscience, Cat. Nr. 563797, clone SK7). Cells were then fixed and also stained with intracellular antibodies IFNγ-FITC (1:10, BD Pharmingen, Cat. Nr. 340449, clone 25723.11). Flow cytometric analysis was performed on the CytoFlex Cell Analyser (Beckman Coulter).

# **2. SDC Tables**

**SDC Table 1**. Selected laboratory values of patients at different time points.

| Patient No. | 1 | 2 | 3 | 4 | 5 | 6 |
| --- | --- | --- | --- | --- | --- | --- |
| G-CSF application | | | | | | |
| - to treat | yes | no | yes | no | no | no |
| - to prevent   ICT-induced neutropenia | no | yes | no | yes | no | no |
| Leukocyte counts [G/L] | | | | | | |
| On admission | 0,15 | 33,2 | 3,7 | 3,3 | 2,8 | 1,4 |
| At time of deterioriation | 3,0 | 5,7 | 3,6 | 6,8 | 3,7 | n/a |
| Absolute neutrophil counts [G/L] | | | | | | |
| At start of G-CSF | <0,1 | 1,8 | <0,1 | 5,6 | n/a | n/a |
| At time of clinical deterioriation | 2,5 | 4,8 | 3,2 | 6,3 | 3,5 | n/a |
| Lymphocyte counts [cells/µL] | | | | | | |
| On admission | 40 | n/a | 620 | 33 | 366 | 85 |
| At time of clinical deterioriation | 30 | 742 | 69 | 136 | 74 | n/a |

# **3. SDC Figures**

**SDC Figure 1**. CONSORT diagram for study cohort.

**SDC Figure 2.** Overview of available peripheral blood samples from B-cell depleted lymphoma patients.

**
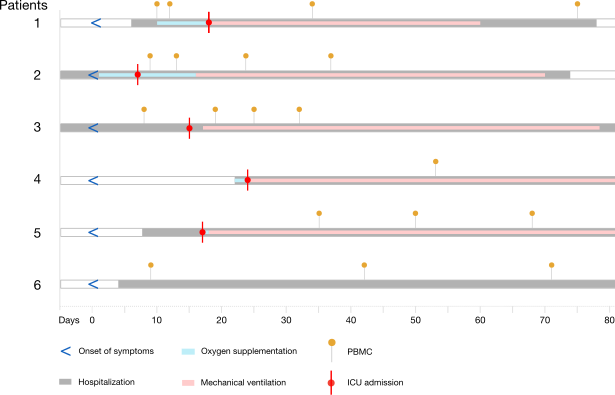
**

**SDC Figure 3.** Gating strategies for **(A)** B-cells (in B-NHL patients), **(B)** B-cells (in controls), **(C)** monocytes, and **(D)** T-cells from PBMCs.

**SDC Figure 4.** **(A)** Unsupervised clustering of patients and controls based on gene expression profiling. Quantification of **(B)** B-cells (B-cell score) **(C)** macrophages (macrophage score) and **(D)** neutrophils (neutrophil score) in control (left) and patients (right) inferred from digital multiplex gene expression data (nCounter, NanoString, nSolver software). **(E)** GSEA for the Hallmark Interferon Alpha Response signature. Changes in the log2 ratios of **(F)** HLA-DRA and **(G)** CXCL5 over time. Day 10 corresponds to respiratory deterioration (time of intubation) and it is used as normalization time point.

**SDC Figure 5.** Dynamic gene expression patterns with peaks at time of respiratory deterioration (intubation) (patient 1 – 3).

**SDC Figure 6.** Dynamic gene expression patterns with troughs at time of respiratory deterioration (intubation) (patient 1 – 3).

**SDC Figure 7.** Heatmap of top 20 up- and down-regulated genes in patients and controls at early time points of COVID-19.

**SDC Figure 8.** Heatmap of differentially expressed genes annotated to the interferon pathway in patients and controls at early time points of COVID-19.

**SDC Figure 9.** SARS-CoV-2 specific IgG levels in blood serum of B-cell depleted lymphoma patients no. 1, 2 and 3 at indicated time points. Dashed lines indicate positivity threshold for IgG levels.

# **4. SDC Reference**

1. Muenchhoff M, Mairhofer H, Nitschko H, et al. Multicentre comparison of quantitative PCR-based assays to detect SARS-CoV-2, Germany, March 2020. *Eurosurveillance*. 2020;25(24):2001057.
